# Prognostic prediction based on histopathologic features of tumor microenvironment in colorectal cancer

**DOI:** 10.3389/fmed.2023.1154077

**Published:** 2023-04-06

**Authors:** Liang Shi, Yuhao Zhang, Hong Wang

**Affiliations:** ^1^School of Clinical Medicine, Hebei University, Baoding, Hebei, China; ^2^The First Department of General Surgery, Cangzhou Central Hospital of Hebei Province, Cangzhou, Hebei, China; ^3^Department of Neurosurgery, Zhejiang Provincial People's Hospital, Affiliated to Hangzhou Medical College, Hangzhou, Zhejiang, China

**Keywords:** tumor microenvironment, stroma, whole slide images, pathway, colorectal cancer

## Abstract

**Purpose:**

To automatically quantify colorectal tumor microenvironment (TME) in hematoxylin and eosin stained whole slide images (WSIs), and to develop a TME signature for prognostic prediction in colorectal cancer (CRC).

**Methods:**

A deep learning model based on VGG19 architecture and transfer learning strategy was trained to recognize nine different tissue types in whole slide images of patients with CRC. Seven of the nine tissue types were defined as TME components besides background and debris. Then 13 TME features were calculated based on the areas of TME components. A total of 562 patients with gene expression data, survival information and WSIs were collected from The Cancer Genome Atlas project for further analysis. A TME signature for prognostic prediction was developed and validated using Cox regression method. A prognostic prediction model combined the TME signature and clinical variables was also established. At last, gene-set enrichment analysis was performed to identify the significant TME signature associated pathways by querying Gene Ontology database and Kyoto Encyclopedia of Genes and Genomes database.

**Results:**

The deep learning model achieved an accuracy of 94.2% for tissue type recognition. The developed TME signature was found significantly associated to progression-free survival. The clinical combined model achieved a concordance index of 0.714. Gene-set enrichment analysis revealed the TME signature associated genes were enriched in neuroactive ligand-receptor interaction pathway.

**Conclusion:**

The TME signature was proved to be a prognostic factor and the associated biologic pathways would be beneficial to a better understanding of TME in CRC patients.

## 1. Introduction

Colorectal cancer (CRC) is the third most common diagnosed cancer and the second leading cause of cancer death (1). Management and treatment of these malignant tumors largely depend on histopathologic diagnosis. Subjective evaluation of histologic slides by experienced pathologists is the gold standard for cancer diagnosis and staging. Molecular and genetic test plays a leading role in the field of quantitative biomarkers (2, 3). Although the tumor node metastasis (TNM) staging system is the basis for treatment decision of CRC patients, different outcomes observed within each stage calls for improved informative markers (4, 5).

Histologic biomarkers focus on the morphological aspects and composition of tumors, rather than their anatomical location and behavior. The substance of the tumor is comprised not only of neoplastic cells but also surrounding stroma which includes immune cells, fibroblasts, signaling molecules and extracellular matrix. These components collectively make up the tumor microenvironment (TME) (6, 7). Survival analyses have demonstrated that valuable aspects of the TME, such as variations in tumor stroma, the presence of tumor budding and host inflammatory response may outperform conventional TNM staging (8–10). Pathologists can recognize these prognostically valuable aspects of the TME, however, the description and quantification of TME is not a routine procedure for pathologists (11). Besides, pathologists visually assessed TME on hematoxylin and eosin (H&E)-stained sections under the microscope, inevitably causing much discrepancies among pathologists. For example, interobserver agreement of tumor-stroma ratio (TSR) assessment ranges from 0.239 to 0.886 (Cohen’s kappa) (12–14). Due to these facts, an automatic TME assessment framework would be valuable, which could lead to better risk stratification, prognosis prediction and treatment support.

The increased availability of digital whole slide images (WSIs) and the successful application of convolutional neural networks (CNNs) in medical imaging, presents an opportunity for fully automatic pathologic assessment of CRC. Kather et al. proposed to use a VGG-based classifier (15) to recognize different components in WSIs of CRC patients, and the intermediate activation of the classifier was proved to be related to survival (16). Zhao et al. further improved the model by use of larger training data and quantified TSR in WSIs (17). Jiao et al. reconsidered the evaluation of TME in colon adenocarcinoma. In addition to stroma component, other tissue types in the tumor mass, especially necrosis and lymphocyte components, are also considered (18). However, the biologic pathways associated with the TME features that stratify patients for prognosis are elusive, which becomes one of the barriers preventing computational histopathology into clinical translation.

Therefore, we not only aim to develop a WSI-based TME signature to predict prognosis in a public dataset but also to explore the biological basis of the prognostic TME signature by revealing key pathways associated with the TME signature that confer prognostic significance in CRC patients.

## 2. Materials and methods

### 2.1. Study design

The overall design of the present study included four steps: tissue segmentation, TME quantification, TME signature development and validation, and pathway/gene identification. First, we developed a deep learning model to identify different TME components on WSI. Second, we calculated some quantitative features to describe the characteristics of TME. Third, we performed survival analysis to assess the prognostic value of the TME features and developed a TME signature for prognosis prediction. Forth, we identified significantly associated genes for annotating individual prognostic TME signature.

### 2.2. Data acquisition

The WSIs, clinical data and genome data supported this study were downloaded from The Cancer Genome Atlas (TCGA) database,^1^ the colon adenocarcinoma project (TCGA-COAD) and the rectal adenocarcinoma project (TCGA-READ). The TCGA-COAD project and TCGA-READ projects are two multicenter cohorts, where 461 and 172 patients involved, respectively. The WSIs were H&E stained diagnostic slides with “.svs” format and gene level expression were measured by RNA sequencing data of upper quartile normalized Fragments per Kilobase of transcript per million mapped reads (FPKM-UQ). The follow-up data were extracted from a published study, namely the pan-cancer clinical data resource of TCGA (TCGA-CDR) (19). The progression-free survival (PFS) information and clinical variables including age, sex, T stage and N stage were extracted from TCGA-CDR for the following survival analysis.

### 2.3. Preprocessing

The WSIs are scanned at 20X (0.5 μm/pixel) or 40X (0.25 μm/pixel) magnification, so that each image can even contain 100,000 × 100,000 pixels. However, most of the regions on WSIs are blank area, which do not contribute to TME quantification. To accelerate the WSIs analysis, we used adaptive Otsu method (20) for rough foreground segmentation at a low resolution of 112 μm/pixel.

According to the genecode_v22_annotation_gene_probeMap document downloaded from the TCGA, we renamed the identifiers of probes to gene symbols, and averaged the gene expression data if multiple probes were mapped to the same gene symbol. Then, we excluded the genes that expressed in less than 20% of samples.

### 2.4. Tissue segmentation

In order to realize automatic quantitative analysis of TME, a robust model for TME components recognition is necessary. Kather et al. had published their tissue type classification model of CRC for free (16). This model classified the WSIs into nine classes: adipose (ADI), background (BACK), debris (DEB), lymphocytes (LYM), mucus (MUC), muscle (MUS), normal colon mucosa (NORM), cancer-associated stroma (STR) and colorectal adenocarcinoma epithelium (TUM). This model was trained on the NCT-HE-100 K dataset and validated on the CRC-VAL-HE-7 K dataset^2^ and used the Macenko stain normalization algorithm (21) for image preprocessing using the MATLAB software. All images of the two datasets were obtained at 20X (0.5 μm/pixel) with a size of 224 pixels × 224 pixels. The model was built based on VGG-19 architecture and used transfer learning strategy which was pretraining the model on Imagenet dataset for parameters initialization. This model achieved an accuracy of 94.3% in the patch-level classification task on the validation dataset. VGG-19 has been proved to have well tissue classification ability and perform better than Alexnet, Googlenet, Resnet50 and Squeezenet by Kather’s study (16).

Because of the commercial software restrictions of the MATLAB,^3^ we retained this model using the open source software Python according to the training configurations of Kather et al. In order to apply the trained TME components recognition model on TCGA-COAD and TCGA-READ WSI dataset, we tiled the WSIs at 20X (0.5 μm/pixel) into unoverlapped patches with the size of 224 pixels × 224 pixels.

### 2.5. TME features

In the previous study of Zhao et al. (17), TSR is defined as a metric of areastroma/(areastroma + areatumor) × 100%. In the present study we extended this metric to other TME components besides the BACK and DEB. Furthermore, we also considered relative ratio of the eight TME components in the foreground content as TME features. Thus, we totally defined 13 TME features for further analyses, of which the metrics are listed in Table 1. The correlation between any two TME features was calculated evaluated by Pearson correlation coefficient.

**Table 1:** Metric of TME features in the present study.

TME feature	Metric
ADI ratio	area_adipose_/area_foreground_ × 100%
LYM ratio	area_lymphocytes_/area_foreground_ × 100%
MUC ratio	area_mucus_/area_foreground_ × 100%
MUS ratio	area_muscle_/area_foreground_ × 100%
NORM ratio	area_normal colon mucosa_/area_foreground_ × 100%
STR ratio	area_stroma_/area_foreground_ × 100%
TUM ratio	area_tumor_/area_foreground_ × 100%
TAR	area_adipose_/(area_adipose_ + area_tumor_) × 100%
TLR	area_lymphocytes_/(area_lymphocytes_ + area_tumor_) × 100%
TMUCR	area_mucus_/(area_mucus_ + area_tumor_) × 100%
TMUSR	area_muscle_/(area_muscle_ + area_tumor_) × 100%
TNR	area_normal colon mucosa_/(area_stroma_ + area_tumor_) × 100%
TSR	area_stroma_/(area_stroma_ + area_tumor_) × 100%

### 2.6. TME signature development and validation

The samples were randomly allocated into a training set and a validation set at a ratio of 7:3. Univariate Cox proportional hazard regression analysis was performed to investigate the association between TME features and PFS in the training set. The significant TME features were then combined by a Cox proportional hazard regression model to generate a TME signature to predict the PFS. The sample were grouped into a high group and a low group by using the median TME signature. Kaplan–Meier survival analysis with logrank test was performed to validate the prognostic value of the TME signature. Then a combined model that combined the TME signature and clinical variables using Cox proportional hazard regression were developed to predict the PFS. The time-dependent receiver operating characteristic (ROC) curves were used to evaluated the combined model. The TME signature and combined model were developed in the training set and evaluated in the validation set. The performance of the TME signature and combined model was further evaluated by 10-fold cross validation.

### 2.7. Gene-set enrichment analysis

Wilcoxon rank-sum test was performed to identify the significantly associated genes with the TME signature. The significant genes with false discovery rate (FDR)-adjusted *p* < 0.05 and |log2(fold change)| value >0.5 were enriched to find significant pathways using R package “clusterProfiler” by querying Gene Ontology (GO) annotation database and Kyoto Encyclopedia of Genes and Genomes (KEGG) database. FDR-adjusted *p* < 0.05 indicated significant enrichment. The significant enriched biologic functions were used to annotate the TME signature.

## 3. Results

### 3.1. Patients characteristics

**Figure 1:**
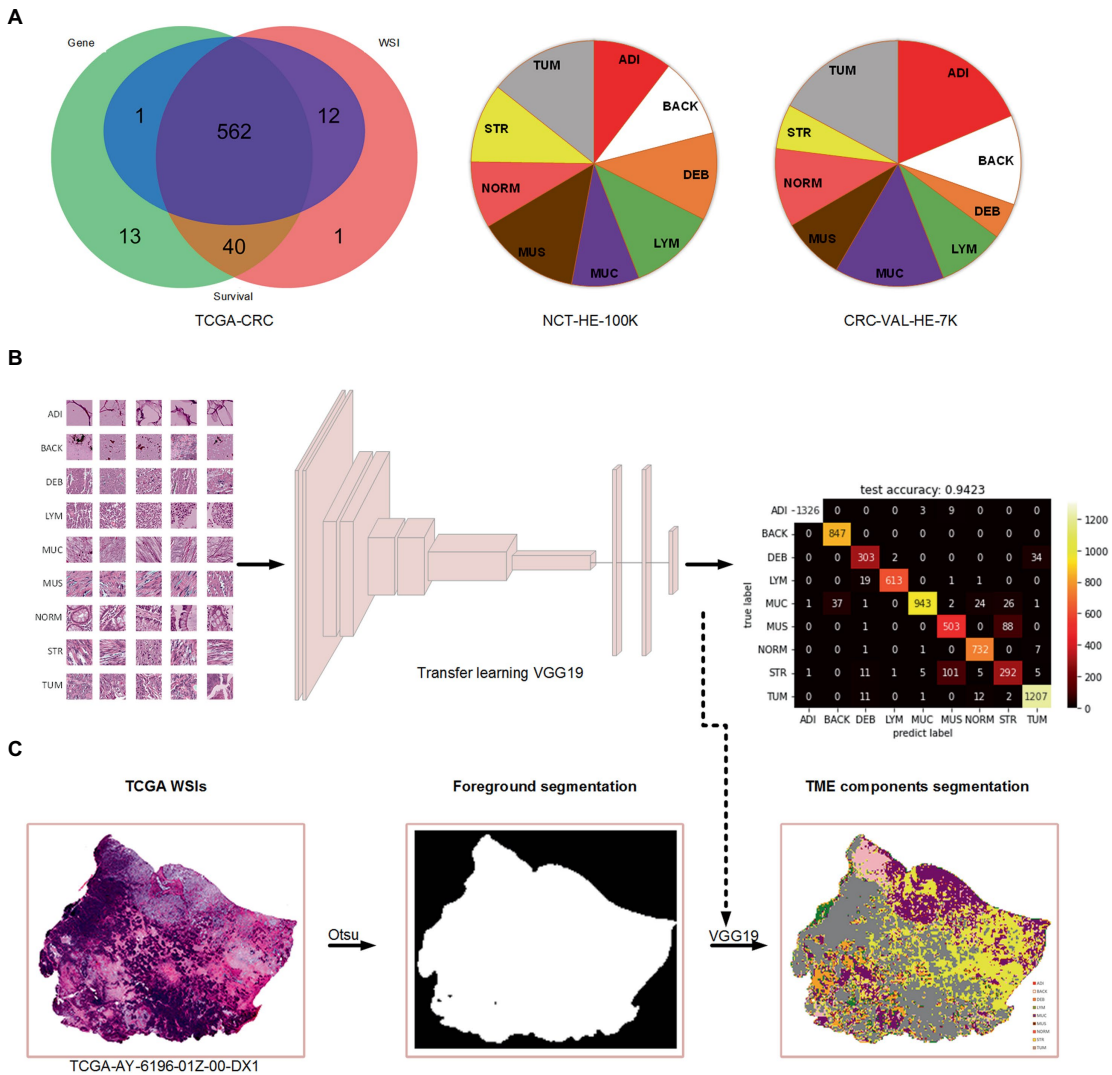
The data used in the present study and the process of TME components recognition. **(A)** Venn diagram of the collected data from TCGA-CRC and the percentage of nine tissue types in two cohorts of Kather et al.’s study. **(B)** Training a VGG19-based tissue recognition model by transfer learning strategy. **(C)** An example of foreground segmentation and TME components segmentation.

A total of 615 patients with 624 diagnostic WSIs were downloaded from the TCGA-COAD and TCGA-READ (hereafter called TCGA-CRC) project. A total of 616 patients with the gene expression data of the primary tumor were selected from the TCGA-CRC. A total of 590 patients with clinical variables and survival information were extracted from TCGA-CDR study, of which 15 patients with a follow-up time less than 30 days were excluded for decreasing the negative effects on accuracy of constructing prognostic models. Finally, 562 patients with WSIs, gene expression, clinical variables and survival information were included for further analyses, as shown in Figure 1A. The demographic information of these included patients are listed in Table 2.

**Table 2:** Demographic information of this study.

Variable	Value
**Age, years**	
Median (interquartile, IQR)	68 (58–75)
Mean (Standard Deviation, SD)	66.2 (12.5)
**Sex**	
Female	259
Male	303
**T stage**	
T1	17
T2	98
T3	388
T4	59
**N stage**	
N0	318
N1	136
N2	108
**Endpoints (uncensored/all)**	
Progression-free survival	148/562

### 3.2. Tissue segmentation

In this study, we used the Otsu method which only costs few seconds (<5 s) per WSI for the foreground segmentation to accelerate the TME components identification. We retained the TME components recognition model on color normalized NCT-HE-100 K dataset and validated it on color normalized CRC-VAL-HE-7 K dataset with an accuracy of 94.2%. The heatmap of prediction confusion matrix on CRC-VAL-HE-7 K dataset is shown in Figure 1B, and an example of TME components identified by the model is shown in Figure 1C. Three patients were found to have no tumor patches on their WSIs, so that the two patients were excluded in the further analyses.

### 3.3. TME signature development and validation

**Figure 2:**
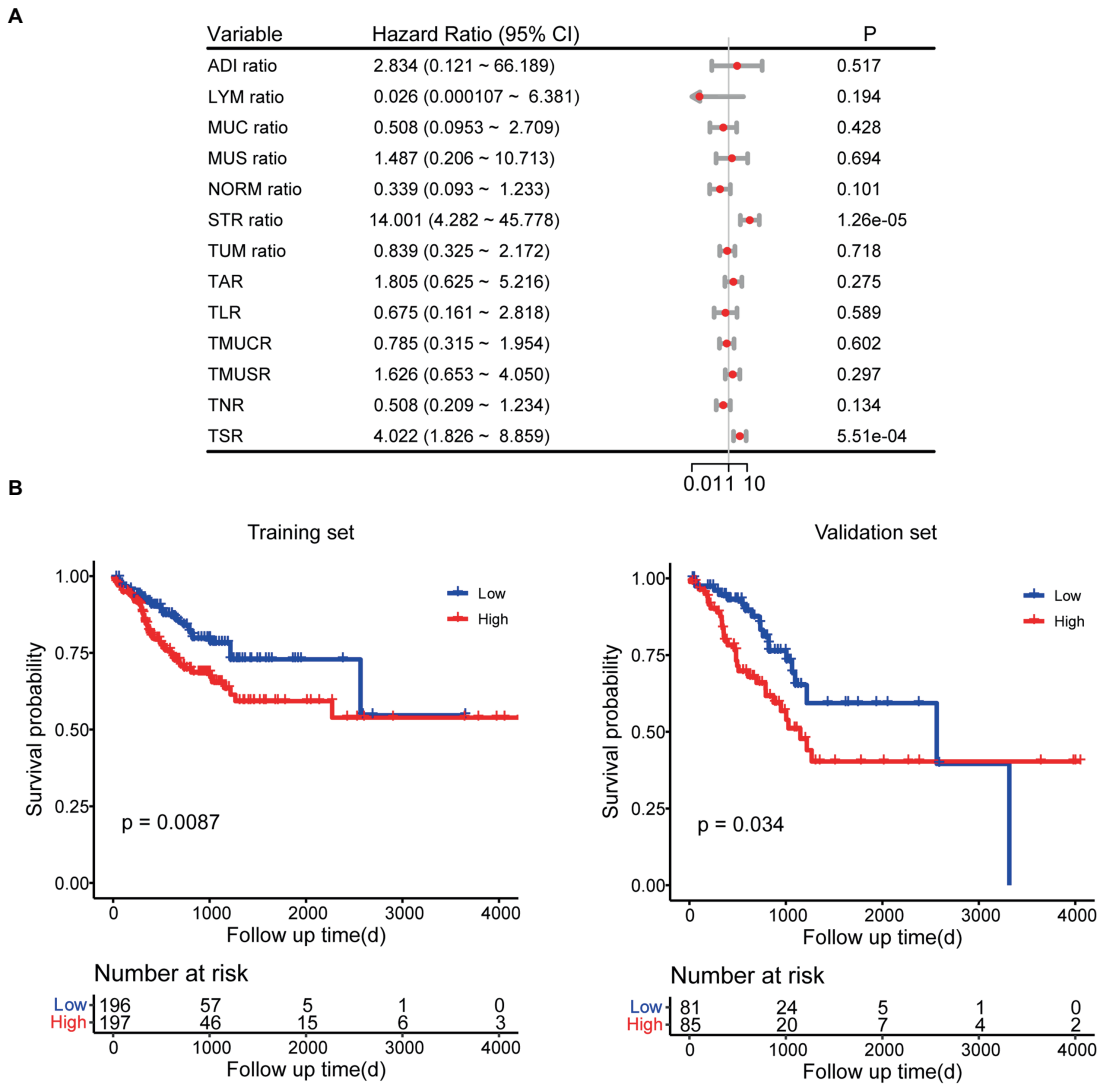
Survival analyses. **(A)** Univariate Cox regression analyses for TME features. **(B)** Kaplan–Meier survival analyses for TME signature.

**Figure 3:**
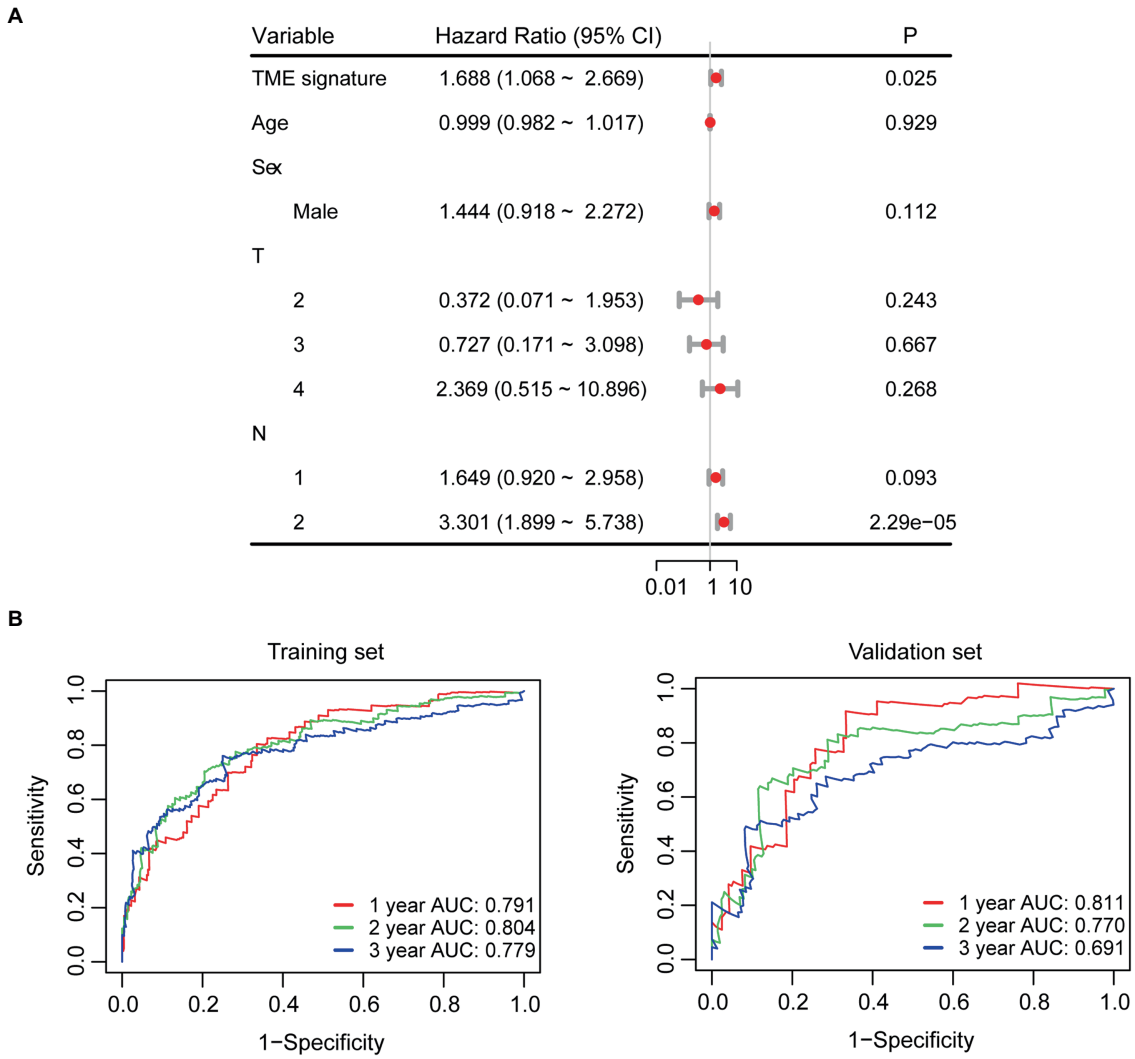
Survival analyses. **(A)** Multivariate Cox regression analyses for TME signature and clinical variables. **(B)** Time-dependent ROC curves of the combined model.

In the univariate Cox regression analyses, STR ratio in foreground content and the TSR were found to be significantly (*p* < 0.05) associated to PFS. The hazard ratio with the 95% confidence interval (CI) of these TME features in Cox regression analyses are summarized in Figure 2A. STR ratio and TSR were found to be highly correlate, because their correlation coefficient achieved 0.860, as shown in Supplementary Figure S1. The concordance index (C-index) of the STR ratio and TSR were listed in Table 3. Compared with TSR, STR ratio achieved better predictive ability. The TME signature that combined the STR ratio and TSR was developed by using Cox regression. The weights and *p* values of the two features for TME signature development are listed in Table 4. The p value indicated STR ratio is more important than TSR for TME signature calculation. The C-index of the TME signature was 0.638 (95% CI: 0.574–0.701) and 0.625 (95% CI: 0.542–0.708) in the training set and validation set, respectively. Kaplan–Meier survival curves of the TME signature with logrank test *p*-values were plotted in Figure 2B. In the multivariate Cox regression analysis, the TME signature was still significantly associated with PFS when considering clinical variables, as shown in Figure 3A. The combined model achieved a C-index of 0.752 (95% CI: 0.702–0.802) and 0.714 (95% CI: 0.642–0.786). Figure 3B showed the time-dependent ROC curves of the combined model for PFS from 1 to 3 years. The result of performance comparison indicated that the TME signature performed better than TSR, as shown in Table 3. Moreover, the predictive ability of signature can be further improved by adding clinical information. The mean C-index of the TME signature and combined model in the cross-validation was 0.613 and 0.711, respectively, as shown in Table 5. The result of cross-validation indicated the TME signature and the combined model both had well predictive ability.

**Table 3:** C-index of different models.

Model	Training set	Validation set	*P*
TME signature	0.638	0.625	-
STR ratio	0.637	0.604	0.136
TSR	0.618	0.576	0.041
Combined model	0.752	0.714	0.012

*P*-values were used for evaluating the performance difference between the TME signature and the other models in validation set.

**Table 4:** Weights of STR ratio and TSR for calculating TME signature.

Variable	Weight	*P*
STR ratio	2.667	0.028
TSR	−0.023	0.979

**Table 5:** C-index of TME signature and the combined model in 10-fold cross-validation.

Fold	TME signature	Combined model
1	0.547	0.630
2	0.575	0.692
3	0.604	0.762
4	0.678	0.751
5	0.613	0.703
6	0.625	0.691
7	0.532	0.610
8	0.723	0.815
9	0.677	0.769
10	0.559	0.685
Mean C-index	0.613	0.711

### 3.4. Gene-set enrichment analysis

**Figure 4:**
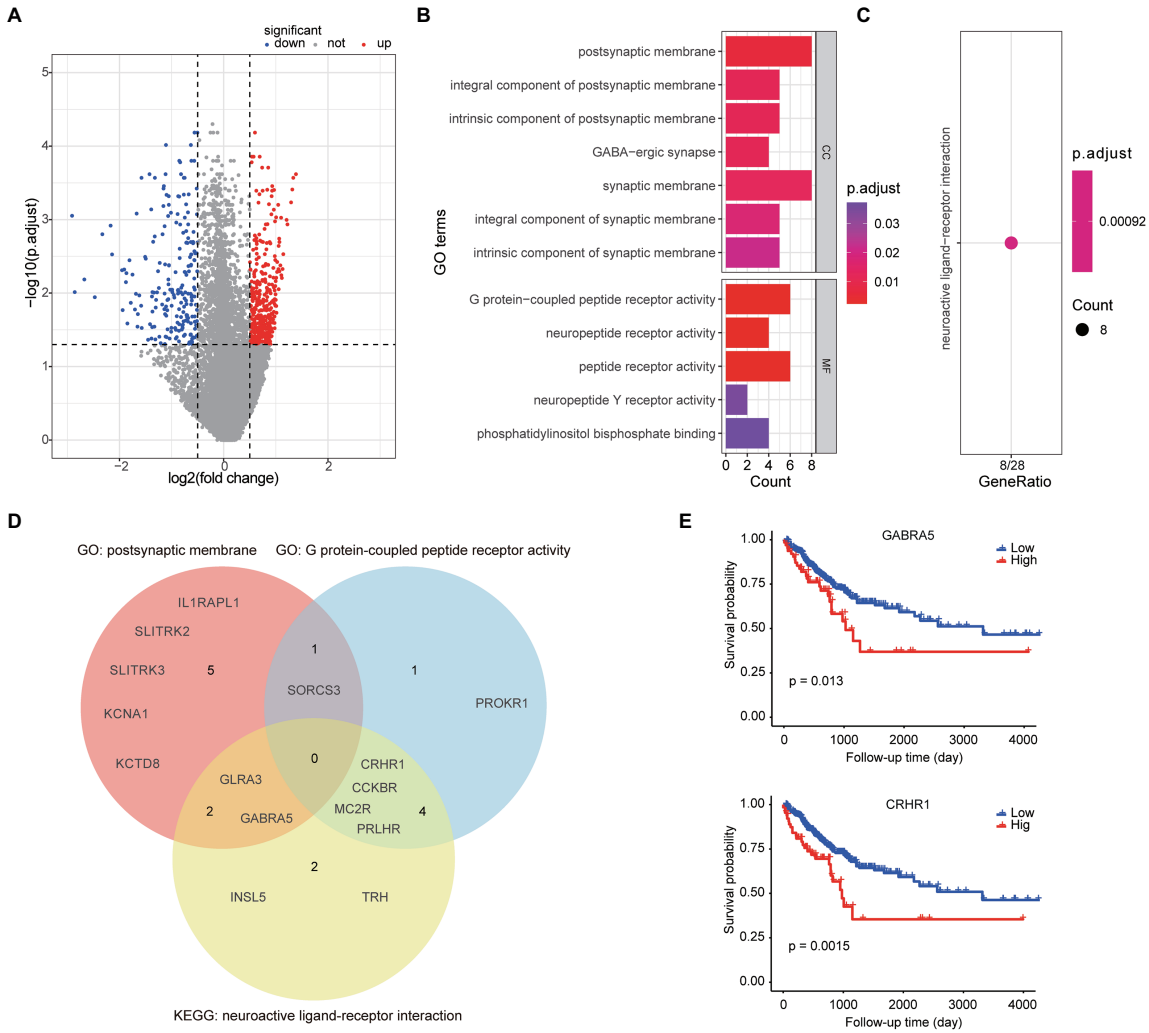
Bioinformatics analyses of TME signature associated biologic basics. **(A)** Volcano plot of gene expression between the high-TME signature group and the low-TME signature group. **(B)** The significantly enriched GO terms. **(C)** The significant enriched KEGG pathways. **(D)** Venn diagram of the gene symbols that involved in two significant GO terms and a KEGG pathway. **(E)** Two genes significantly associated to PFS.

A total of 595 genes were found to differently expressed between high- and low-TME signature groups by using rank-sum test corrected by FDR. Compared to the low group, 380 genes were up-regulated and 215 genes were down-regulated, as shown in Figure 4A. Only 44.2% of the 595 genes were successfully mapped to ENTREZID in GO database. The GO analysis revealed that these TME signature associated genes were enriched in postsynaptic membrane related function and G protein-coupled peptide receptor activity function as shown in Figure 4B. The KEGG analysis revealed that these genes were enriched in neuroactive ligand-receptor interaction pathway (Figure 4C). The genes involved in the significantly related GO terms and KEGG pathways were shown in Figure 4D. The Kaplan–Meier survival curves of the genes enriched in the significant GO terms and KEGG were plotted in Figure 4E.

## 4. Discussion

In this study, we presented a workflow of automatic TME quantification on WSIs by using deep learning model, and developed a significant TME signature for PFS prediction. We found that the TME features STR ratio and TSR were significant prognostic factors of PFS in CRC patients. The developed TME signature that combined the STR ratio and TSR still had well predictive ability, when considering clinical variables. The combined model could further improve the prediction. We further annotated the TME signature by gene-set enrichment analysis and found some significantly associated genes were enriched in postsynaptic membrane related function and G protein-coupled peptide receptor activity function. *GABRA5* and *CRHR1*, the TME signature associated genes belong to postsynaptic membrane activity function gene-set and G protein-coupled peptide receptor activity function gene-set respectively, were identified as prognostic factors by Kaplan–Meier survival analysis.

It has been reported that TME has a great correlation with the occurrence, development and prognosis of CRC (22, 23). Stromal cells are the major the non-tumor component of TME, which play important roles in evolution of cancers. Recent literature about the TME has shed light on CRC tumorigenesis and the complex interactions between tumor cells and the surrounding stroma (24, 25). Tumor-associated stroma is composed primarily of tumor-associated fibroblasts and extracellular matrix, whose extensive characteristics have been identified relevant roles in promoting tumor growth and invasion (26), angiogenesis (27), and energy homeostasis (28). Therefore, the quantification of TME in CRC might help us formulate a more sensible management and treatment plan for CRC patients. Zhao et al. has developed a deep learning-based TSR using their private data and demonstrated the TSR was significantly associated with OS in CRC patients (17). In our study, the Cox regression analysis showed the TSR was also a significant prognostic factor for PFS. The use of OS as an endpoint may undermine clinical studies, because noncancer causes of death do not necessarily reflect tumor biology, invasiveness, or response to treatment. Thus, in consideration of the relatively short follow-up time of TCGA-CRC cohort, we used PFS as an endpoint. Our study revealed that a high STR ratio in TME or high TSR is associated with the poor PFS. STR ratio has more prognostic power than TSR, a well-established signature. Combining STR ratio and TSR would generate a better performed signature. However, these results remain to be validated in other cohorts.

Based on the developed TME signature, we further explored the mechanism associated with the high- and low-TME signature groups. The TME signature associated genes were mainly enriched in Go terms called postsynaptic membrane and G protein-coupled peptide receptor activity. More interesting, both the *GABRA5* and *CRHR1* genes that belong to two different GO terms, were not only significantly associated with PFS but also belong to the same pathway called neuroactive ligand-receptor interaction. Neuroactive ligand-receptor interactions have been shown to be associated with other gastrointestinal cancer (29). Yao et al. found that the neuroactive ligand-receptor interaction is significantly associated with the development of colorectal cancer according to the GO and KEGG enrichment analyses (30). Whether neuroactive ligand-receptor can directly regulate the formation of stroma and how to further affect tumor progression in colorectal cancer are worthy of further exploration. Develop appropriate regulatory drugs targeting relevant pathways may contribute to the treatment of CRC.

The present study has some limitations. First, our study only included the TCGA-CRC cohort, other independent cohorts are needed for validation of our findings. Second, the prediction accuracy of deep learning model for recognition of TME components should be improved, to ensure the calculated TME features are closer to the real situation. Some new models such as PDBL (31), CRCCN-Net (32) and Vision Transformer (33) are worthy of being using, since they have achieved accuracy of more than 96% in Kather’s dataset. Third, nearly half TME signature associated genes failed to be identified by GO and KEGG databases, which may affect the comprehensiveness of functional annotations for TME signature.

In conclusion, the present study validated the feasibility and validity of using deep learning model to quantify the TME, and developed a TME signature for survival prediction. The stroma ratio significantly related to prognosis in CRC was proved once again. The TME signature associated biological pathways were also preliminarily explored. We believe that these findings will be beneficial to the treatment and management of patients with CRC.

## Data availability statement

The datasets presented in this study can be found in online repositories. The names of the repository/repositories and accession number(s) can be found in the article/Supplementary material.

## Ethics statement

The studies involving human participants were reviewed and approved by the Ethics Committee of Hebei University. Written informed consent for participation was not required for this study in accordance with the national legislation and the institutional requirements.

## Author contributions

LS and HW contributed to conception and design of the study. LS and YZ organized the database and performed the statistical analysis. LS wrote the first draft of the manuscript. LS, YZ, and HW wrote sections of the manuscript. All authors contributed to the article and approved the submitted version.

## Conflict of interest

The authors declare that the research was conducted in the absence of any commercial or financial relationships that could be construed as a potential conflict of interest.

## Publisher’s note

All claims expressed in this article are solely those of the authors and do not necessarily represent those of their affiliated organizations, or those of the publisher, the editors and the reviewers. Any product that may be evaluated in this article, or claim that may be made by its manufacturer, is not guaranteed or endorsed by the publisher.

## Footnotes

^1^https://portal.gdc.cancer.gov

^2^http://dx.doi.org/10.5281/zenodo.1214456

^3^https://www.mathworks.com/
