# Supplementary figures and images for: Prognostic prediction based on histopathologic features of tumor microenvironment in colorectal cancer
Source: Front Med (Lausanne). 2023 Apr 6;10:1154077. doi: 10.3389/fmed.2023.1154077 (PMC10117979; doi:10.3389/fmed.2023.1154077)

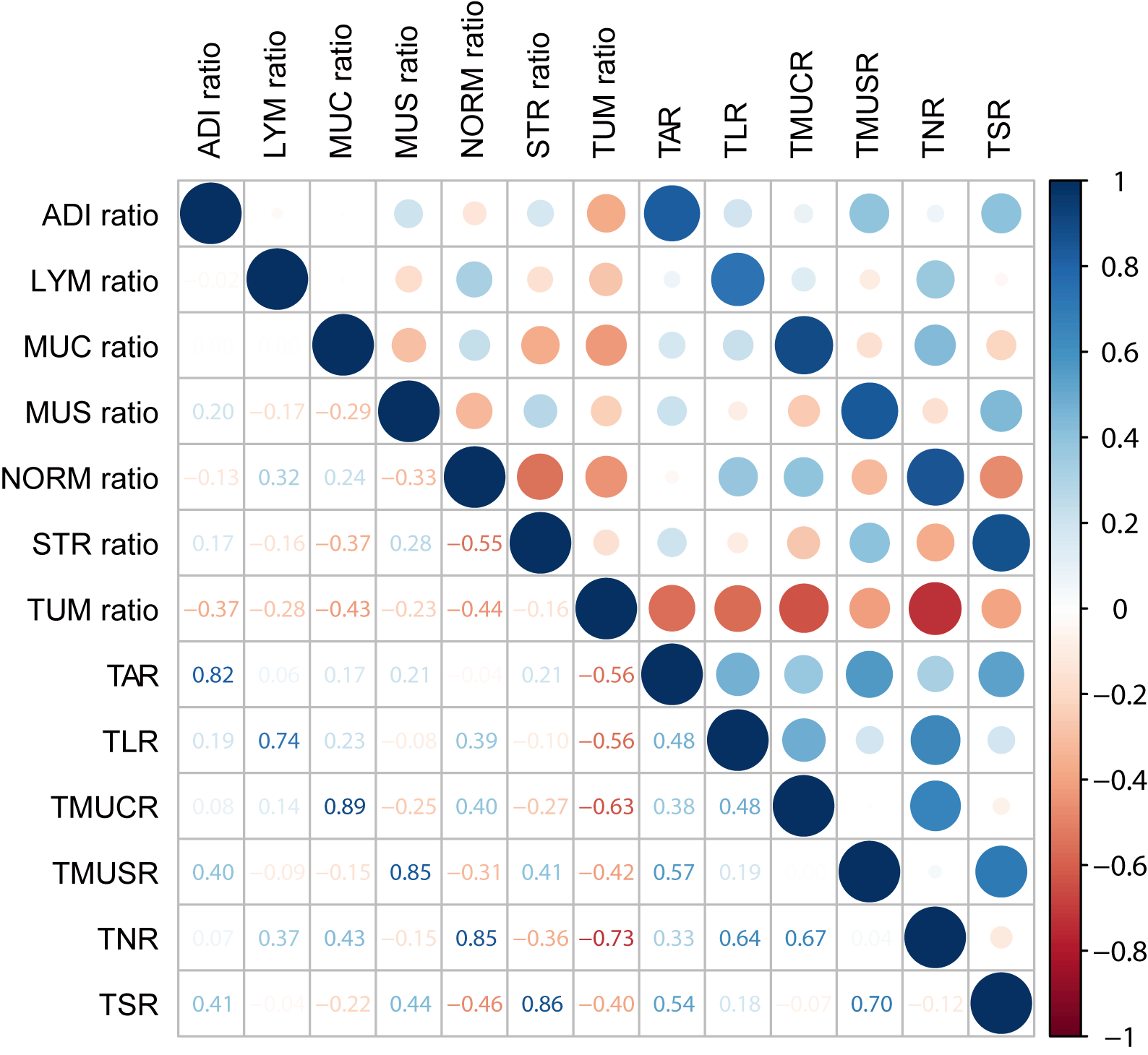

Supplement: Supplementary file 1 [file Image_1.TIF]
